# Transmission status of lymphatic filariasis in hotspots of filarial infection, persistent districts of nepal during post-MDA surveillance

**DOI:** 10.1371/journal.pone.0338141

**Published:** 2026-01-16

**Authors:** Pramod Kumar Mehta, Mahendra Maharjan

**Affiliations:** Central Department of Zoology, Institute of Science and Technology, Tribhuvan University, Kirtipur, Nepal; University of Energy and Natural Resources, GHANA

## Abstract

**Background:**

The lymphatic filariasis (LF) elimination program in Nepal was launched in 2003, three years after the implementation of a global program to eliminate lymphatic filariasis globally. Based on antigenic prevalence below the cut-off value, i.e., ≤ 2% shown by the transmission assessment survey (TASI) report of 2017–2018, the program was stopped in Nepal. The report indicated that antigen-positive children were clustered in the community and considered hotspots. In order to find the status of lymphatic filariasis transmission at the community level, the present study was designed in the hotspots to assess antigenemia among the children born after mass drug administration (MDA), along with vector infection/infectivity in identified hotspots of Central Nepal.

**Methods:**

Each of two districts from the hilly and Terai regions of Central Nepal was selected based on comparatively high antigenic prevalence shown by previous TASI reports, and a descriptive cross-sectional study was designed. Two specific methods were employed: a Filarial Test Strip (Alere, Scorborough, ME) was used for antigenemia (n = 791) among children, and gravid traps were used for vector mosquito collection. Parous mosquitoes (n = 3344) were dissected for infection/or infectivity.

**Results:**

The result indicated that antigen-positive cases were unexpectedly increased in hotspots of one each from the hilly region (Dhading) and Terai region (Mahottari), i.e., above the critical level (≥ 2%). Antigen prevalence increased from baseline prevalence, which was significantly associated with the number of MDA rounds but not with MDA coverage. The upper confidence limit of antigenemia and Mf infection was above the critical cut-off value in hotspots of all selected endemic districts. However, none of the vector mosquitoes, such as *Culex quinquefasciatus,* was found to be infected with any larval stage of filarial nematode.

**Conclusions:**

The result showed an increase in antigenemia and Mf prevalence in high baseline prevalence areas with fewer MDA rounds. Lack of correlation between filarial infection in humans and xenomonitoring could be low sensitivity of xenomonitoring by microscopy. TAS-based assessment of antigenemia in children for stopping MDA can be supplemented with molecular xenomonitoring in hotspots.

## Introduction

Lymphatic filariasis is a neglected tropical disease, also known as elephantiasis, which is caused by a group of filarial nematodes and transmitted by different species of mosquitoes to human beings [1]. *Wuchereria bancrofti* is the most common causative agent of LF, followed by *Brugia malayi* and *Brugia timori* [2]. To interrupt the transmission of LF, the World Health Organization (WHO) launched the global program to eliminate lymphatic filariasis (GPELF) in 2001 globally and recommended 5–6 rounds of annual or biannual mass drug administration (MDA) to community individuals of endemic areas [3,4].

Globally, more than 80 countries were endemic with LF, but recently the infection rate has been reduced by 74% while over 657 million people, in 39 countries, require further rounds of MDA [5]. Moreover, around 36 million people are affected with chronic clinical manifestations of LF [6], so GPELF aimed to eliminate LF as a public health problem by 2030 [7]. In 2019, the WHO estimated that the global public health burden of LF was 1.63 million disability-adjusted life years (DALYs) [8]. The highest burden of LF, i.e., approximately 844 million individuals, is still at risk of infection in nine out of eleven South East Asian endemic countries, while 70% of the global burden occurs in India, Bangladesh, Indonesia in Asia, and Nigeria (In Africa) [9].

Nepal is one of the endemic countries in which 63 out of 77 districts were considered to be endemic with LF, and 25 million people were at risk of infection [10]. Due to those public health issues, the Nepal government formulated a national task force to eliminate LF by 2020 [11]. It started the MDA program in 2003 by using a combination of diethylcarbamazine citrate (DEC) and albendazole anti-filarial medicine from the Parsa district of Central Nepal. In 2013, the country had achieved 100% geographical coverage of all endemic districts with the MDA program [12].

Moreover, six rounds of MDA were completed in 2016 in all endemic districts of Central Nepal; however, all endemic districts of the country were covered by 2018 [12]. During 2017–2018, a transmission assessment survey was completed in Central Nepal to monitor the program’s impact. School-based TAS report showed CFA prevalence below the recommended critical cut-off value of WHO (≤ 2%), so the MDA program was stopped in all endemic districts of Central Nepal, but in some foci of districts, with antigen-positive children were clustered in the community [12]. Based on the TAS report 2017–2018, a comparatively high number of antigen-positive cases clustered in a community in which two districts of two different geographical regions (hilly and Terai) were selected for study (Table 1).

**Table 1:** Circulating filarial antigen (CFA) positivity in children during TAS assessment of hotspots in four districts of Nepal.

Region	Districts	Population	LF antigen prevalence (%)												
			Baseline prevalence		MDA		Pre-TAS		TAS Ⅰ			TAS Ⅱ		TAS III	
			Year	Prevalence	Year of started	No. of rounds	Year	Prevalence	Year	Prevalence	No. of Positives	Year	Prevalence	Year	No. of Positives
Hilly	Lalitpur	548,401	2008	1.06	2010	8	2016	0.66	2017	0.21	5^**#**^	2019	0.18	2021	2^**#**^
	Dhading	322,751	2001	14.7*	2007	6	2012	0.8	2013	0.8	9^**ᵦ**^	2017	0.41	2019	3^**ᵦ**^
Terai	Bara	743,950	2001	0.6	2007	11	2012	0.0	2013	1.2	6^**γ**^	2017	1.2	2019	2^**γ**^
	Mahottari	705,838	2001	2.43*	2007	6	2012	0.0	2013	0.6	8^**α**^	2017	0.7	2019	17^**α**^

*Antigen prevalence above the critical cut-off value (≤ 2%). ^**#**^ Dukuchhap and Bungmati of Lalitpur district, ^**ᵦ**^Salyantar of Dhading district, ^**γ**^Ammadar and Khairawa of Bara district, ^**α**^ Matihani of Mahottari district.

Our previous publication focused on microfilaremia in adult individuals who were eligible during the MDA campaign, a standard questionnaire survey for MDA compliance, and the observation of chronic clinical manifestations, such as hydrocele and elephantiasis, in study participants [10]. The current study mainly focused on antigenic survey in children who were born after MDA and entomological survey for infection/and or infectivity of vector mosquitoes to understand the transmission status of LF infection during post-MDA surveillance.

## Methods

### Ethical consideration and consent to participate

The protocol was approved by the Nepal Health Research Council (NHRC/Reg.no. 629/2018). Investigators explained the study’s objective to participants (or their parents/guardians) in the local language, and written consent was obtained from patients/guardians.

### Study area and period

In the areas with persistent infection based on the TASI report, antigen-positive school children were clustered in the community of the village, which was considered a hotspot, and those schools were located within the village. The risk of infection in such hotspots was investigated to determine the transmission status of LF. Based on the TAS report, comparatively high CFA prevalence in two districts from the hilly region, i.e., Lalitpur (0.21%) and Dhading (0.41%) and two districts from the Terai region, i.e., Bara (1.2%) and Mahottari (0.7%) districts of Central Nepal were selected for the study. MDA was stopped in these districts due to antigen prevalence below the WHO-recommended critical cut-off value, i.e., ≤ 2%. It analysed the antigen-positive cases and their cluster in the community of selected districts. We identified CFA clusters in Salyantar of Dhading district (nine cases), Bungmati and Dukuchhap (local name) of Lalitpur district (five cases), similarly Ammadar and Khairawa of Bara district (six cases) and Matihani of Mahottari district (eight cases). In those particular selected areas, individuals were investigated for CFA in children born after MDA and who had never participated in an MDA program, microfilaremia survey in adult individuals who were eligible for participation during MDA and vector survey for infection/or infectivity with *W. bancrofti*. All these areas were investigated in-between May 2019 to April 2022.

### Study population and sampling

We used the Geographical Information System (GIS) data from the Department of Urban Development and Building Construction (DUDBC) [13] of Nepal. Our target sample was 5–14-year-old children who were born after the MDA program for the antigen survey and above 14 years of community adult individuals for the MF survey. The required sample size for antigen, MF, and vector survey from hotspots of each selected district was determined by using the following formulae:

#### Sample size for Antigen survey:.

Children aged 5–14 years were selected for antigen detection.

Overall antigen prevalence was 13% in Nepal.

Using the sample size calculation formula, n = Z^2^pq/ L^2^

n= (1.96)^2*^0.13*0.87/ (0.05)^2^ =174

Non response rate = 10% = 174*10/100 =17.4

So the actual number of samples for antigen detection was (n) = 174 + 17.4 = 191.4 ~ 192

So at least 192 samples were collected from hotspots of each selected district.

#### The sample size determination for mosquito collection.

The sample size was determined based on the expected level of vector infection, i.e., 1.0% [14]. A minimum of 5000 mosquitoes were targeted to collect from hotspots of all four districts of Central Nepal. Around selected houses, gravid traps were kept for the collection of mosquitoes. The house for keeping the trap was selected by systematic random sampling. All collected mosquitoes were dissected and examined for the presence of all stages of parasite (Mf, L1, L2, and L3), which were used for the calculation of infection. In contrast, L3 was used for the infective stage larvae.

We assumed at least five target group populations in each household (hh), so a total of 724 households were selected from the study areas based on a systemic sampling method. A total of 186 and 174 households from each hotspot of Lalitpur and Dhading districts of the hilly region were selected, respectively, for study; similarly, from each hotspot of Bara and Mahottari districts of the Terai region, 141 and 223 households were selected, respectively, to obtain the required sample size. To locate each house, ArcGIS software was used for the antigen and trapping location of mosquitoes.

The selected households were visited door-to-door, and all eligible individuals were enlisted for the required population from each district. A total of 1117 children from 724 households were enumerated for the antigen survey. Individuals who were outside the station during the study and those who didn’t want to participate in this study willingly were excluded. Finally, a total of 791 children of the age group 5–14 years were tested for filarial antigenemia using Filarial Test Strip (Alere, Scarborough, ME) based on their availability, along with obtaining their assent and their parent’s permission.

### LF prevalence, disease burden, and MDA compliance with LF infection status

#### Circulating filarial antigen test.

Consented community children of the age group 5–14 years were examined for *W. bancrofti* CFA using the Filarial Test Strip (Alere, Scorborough, ME) following the manufacturer’s instructions. Briefly, 75 μL of finger-prick blood was collected from each individual and applied to the sample application pad, and a single operator read the results after 10 minutes as positive, negative or undetermined (S1 Fig).

S1 Fig: A positive test result at 10 minutes.

#### Night blood sampling for MF prevalence.

Community children were screened for CFA during the daytime, but a night, blood samples were collected from consented community adult individuals of age group 15–87 years between 10 PM and 4 AM for the MF test. About 60 μL of blood was drawn into three portions of the single slide (S2 Fig).

S2 Fig: Night blood collection by finger finger-prick method for the MF study in the field.

Each drop of blood was spread with the edge of the spreader to make a thick blood smear. The blood smears were air-dried for 12–24 hours and transported to the laboratory for further analysis. By using a diamond pencil, slides of blood smears were marked in the corner and dehaemoglobinized with distilled water for about three minutes, air dried, fixed with acid-alcohol (98 parts of methyl alcohol + conc. HCL 2parts) and stained with Giemsa stain for 1–2 minutes (S3 Fig).

S3 Fig: Blood smear staining in the laboratory.

Air-dried smears were examined under a compound microscope using a 10X objective and confirmed with a 40X objective (S4 Fig).

S4 Fig: A stained slide in a slide box.

**Fig 1:**
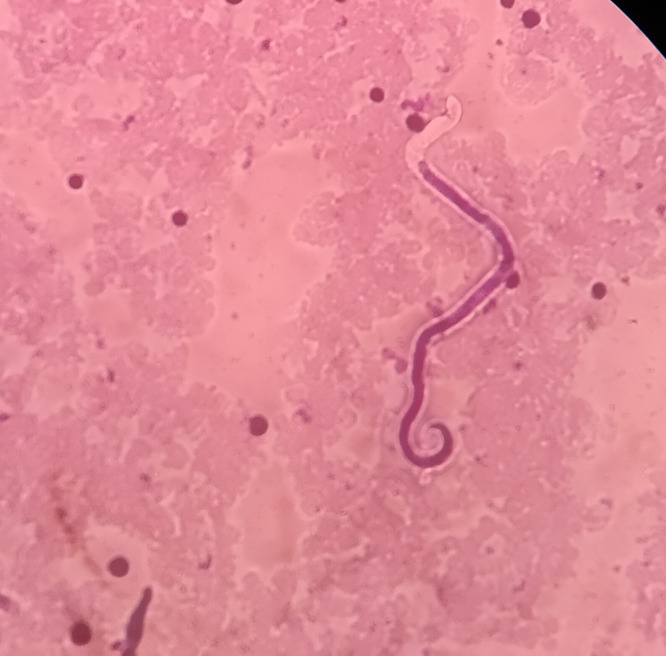
A microfilaria with *W. bancrofti* showing in a blood film.

The results and number of MF were entered against the ID number in the log book. All positive slides and 10% of negative slides were cross-examined by experts. All the LF-positive cases were treated with a standard dose of DEC and albendazole under the supervision of an authorized medical officer (Fig 1).

#### MDA compliance and clinical examination.

By using the pre-designed forms of demographic information were recorded for all participants. All selected individuals were interviewed about whether they had swallowed the DEC and albendazole in at least any one previous round of MDA and the last round of MDA. Based on the standard case definition of chronic clinical manifestations such as hydrocele and elephantiasis of all study participants (age group of 5–87 years) were examined privately by an authorized medical officer (S4 Fig).

S4 Fig: A patient with chronic clinical manifestation (elephantiasis).

### Entomological assessment for vector infection/infectivity

#### Vector sampling.

For xenomonitoring purposes, areas with clusters of antigen-positive cases were selected purposively for placing the gravid traps. A total of 180 gravid traps were placed around the selected households of four hotspots. Vector mosquitoes were collected by using gravid traps, which were standardized in the field concerning the site of placement, appropriate time, and attractiveness of the infusion. Sites proximal to the breeding grounds, the time between 6.00 PM and 6.00 AM and three days old hay infusion could provide maximal yield of vector mosquitoes. Accordingly, the traps were set in the selected sites and consent for the placement of traps was obtained from the family head of the house.

The principle components and architecture of the trap have already been described elsewhere [15]. The infusion was prepared before starting the survey; small pieces of about 200 g of hay were mixed with 45 litres of water in the jar. Mixed 2 gm of malt powder with 2gms of yeast in one litre of water and added to the jar. The jar was closed with a cover and allowed to ferment for 6 days, which could be used for 6–11 days. Three litres of infusion could be required for each gravid trap, and once used was discarded while fresh infusion was used each time. The six gravid traps were placed for 30 days in 180 selected households. The sample size was determined based on the expected level of vector infection (1%). The traps were placed in the evening, and the bag was removed the following morning. The bag was brought to the laboratory. The traps were transported, and adequate care was taken to maintain the mosquitoes alive. In the laboratory, the trapped mosquitoes were removed by using a mechanical aspirator. The female gravid mosquitoes of *Cx. quinquefasciatus* were identified and separated into test tubes while labelled with trap number along with the date of collection. Per-trap density was calculated based on the number of vector mosquitoes collected and the number of active traps used (S5 Fig).

S5 Fig: A collection of mosquitoes by a gravid trap.

#### Detection of filarial parasites in mosquitoes.

Collected mosquitoes from the gravid traps were dissected on the same day. The observations were maintained separately for each trap and per collection. A modified method was followed for the dissection of mosquitoes and examined for infection with the filarial parasite [16]. Mosquitoes collected in the test tube from the traps were anaesthetized by chilling with formalin for a few minutes. Placing individual mosquitoes on a Petri dish, the wings and legs were removed using two pairs of needles or forceps with low-power magnification under a stereoscopic dissecting microscope. An individual mosquito was transferred onto a clean microscope slide and divided into head, thorax, and abdomen using dissecting needles. Each part of the body was placed in a separate drop of saline solution on the same slide. The body parts were further teased into pieces using dissection needles. Care was taken not to break the parasite. Each part of the body was examined for the presence of filarial parasites. The mouth parts were separated with fine needles to allow L3 larvae to escape. The teased parts of the body were examined under a binocular microscope at 10x magnification. When a parasite was noted, the slide was covered with a cover slip and examined at 40x under a compound microscope to count the larvae and their states. The location and number of worms in each body section were noted. The filarial larvae in the infected mosquitoes were categorized into microfilariae (mf), I stage (L1), II stage (L2), III stage (L3), i.e., infective stage.

Mosquitoes carrying any stage of the filarial parasite (MF, L1, L2, or L3 larva) were defined as ‘infected’, while those carrying only L3 larva were defined as ‘infective’. The number of each stage larvae in the infected mosquitoes was counted and recorded.

#### Data analysis.

Exploratory data were entered in Excel spreadsheets (Microsoft Excel 2013) and subsequently analyzed with Minitab 17 version 19.2.0 software. Blood test results for CFA, the presence of hydrocele and elephantiasis, and demographic characteristics were compared by using the Chi-square test and Fisher’s exact test. The lower and upper limits of the 95% CI for the prevalence of CFA and the proportion of female mosquito species were calculated. The sex ratio of mosquitoes was calculated by using a sample proportion test.

## Results

### CFA assessment in selected endemic districts among children born after MDA

#### Lalitpur district.

A total of 176 individuals in the age group of 5–14 years old children were examined for CFA in hotspots of Lalitpur district. The overall male-to-female ratio was 1:1, and the mean age of the participants was 9.1 years. Only one of the children was positive with Ag (0.6%) at the age of six years, which was migrated from other LF endemic districts of Nepal (Table 2).

**Table 2:** Age and gender-wise CFA prevalence in children in selected endemic districts of Central Nepal.

Characteristics		Eligible population(*)	Sampled population(*)	Missed population	No. (%) with CFA [95%CI]	P-value
Lalitpur district						
Age groups in years (n = 176)	5-9	245 (65.3)	74 (30.2)	171 (69.8)	1(1.4) (0.03-7.3)	0.420
	10-14	130 (34.7)	102 (78.5)	28 (21.5)	0 (0.0) (0.0-0.03)	
Gender (n = 176)	Male	235 (62.7)	108 (46.0)	127 (54.0)	1 (0.9) (0.0-0.05)	1.000
	Female	140 (37.3)	68 (48.6)	72 (51.4)	0 (0.0) (0.0-0.04)	
Dhading district						
Age groups in years (n = 202)	5-9	112(53.9)	108(96.4)	4(3.6)	10(9.3)(3.8-14.8)	0.767
	10-14	96(46.1)	94(97.9)	2(2.1)	10(10.6)(4.5-16.9)	
Gender (n = 202)	Male	109(52.4)	105(96.3)	4(3.7)	12(11.4)(5.3-17.5)	0.493
	Female	99(47.6)	97(98.0)	2(2.0)	8(8.2)(2.8-13.7)	
Bara district						
Age groups in years (n = 211)	5-9	135(59.5)	124(91.9)	11(8.1)	2(1.6)(0.2-5.7)	0.513
	10-14	92(40.5)	87(94.6)	5(5.4)	0(0.0)(0.0-3.4)	
Gender (n = 211)	Male	126(55.5)	114(90.5)	12(9.5)	0(0.0)(0.0-2.6)	0.210
	Female	101(44.5)	97(96.0)	4(4.0)	2(2.1)(0.3-7.3)	
Mahottari district						
Age groups in years (n = 307)	5-9	160(52.1)	92(57.5)	68(42.5)	10(10.8)(5.3-19.1)	0.494
	10-14	147(47.9)	110(74.8)	37(25.2)	16(14.6)(8.6-25.5)	
Gender (n = 307)	Male	145(47.2)	105(72.4)	40(27.6)	17(16.2)(9.7-24.7)	0.197
	Female	162(52.8)	97(59.9)	65(40.1)	9(9.3)(4.3-16.8)	

*Symbols indicate the percentage of the samples out of the total in each age class and gender. CFA, Circulating filarial antigen; CI, Confidence interval; P-values are based on Chi-squire analysis and Fisher’s exact test

#### Dhading district.

A total of 202 children of 5–14 years were examined for CFA in the hotspot of Dhading district. The overall male-to-female ratio was 1:1, and the mean age of the participants was 9.1 years. A total of 20/202 (9.9%) had CFA positive, while the antigen infection was not cooperatively varied between males and females (x2 = 0.470, df = 1, P = 0.493). The upper confidence interval of CFA prevalence was greater than the critical cut-off value of the WHO to stop MDA in selected hotspots of Dhading district (Table 2).

#### Bara district.

A total of 211 children of 5–14 years old were examined for CFA, hydrocele and elephantiasis in a hotspot of the Bara district. The overall male-to-female ratio was 0.9, and the mean age of the participants was 8.8 years (range of 5–14 Years). Of 211 individuals screened for LF infection, 2(1%) had CFA positive (Table 2).

#### Mahottari district.

A total of 202 children in the age group 5–14 years were sampled in the Mahottari district for CFA. The overall male-to-female ratio was 1:1, and the mean age of the participants was 10.1 years (range of 5–14 Years). Among them, 26(12.9%) were found to be CFA positive (Table 2).

### Xenomonitoring of LF vectors in selected endemic districts of Central Nepal

#### Lalitpur district.

A total of 1365 mosquitoes were collected by using CDC gravid traps, of which 1175 (86.1%) were *Culex quinquefasciatus,* 190 (13.9%) were non-filarial other mosquitoes such as *Culex vishnui*, *Culex pseudo vishnui,* and *Anopheles* mosquitoes were identified. *Culex quinquefasciatus* mosquitoes were checked for parity in the laboratory, and 979 parous vectors were examined under a microscope for infection with *W. bancrofti*. None of the examined mosquitoes were found infected with *W. bancrofti* larvae of any stage (Table 3).

**Table 3:** Mosquitoes collected by gravid traps (n = 180) and the results of infection and infectivity by microscopy in the selected districts.

Species	Total mosquitoes collected (% out of total)	Male	Female	Proportion of females (95%CI)	Sex ratio, p-value	Total mosquitoes (Per trap density)	Total dissected (%)
Lalitpur district							
*Culex quinquefasciatus*	1175 (86.1)	196	979	0.83 (0.81-0.85)	<0.001	50/1175(23.5)	979 (83.3)
*Culex Vishnui*	85 (6.2)	22	63	0.74 (0.63-0.83)	<0.001	50/85 (1.7)	–
*Armigeres spp.*	92 (6.8)	25	67	0.73 (0.63-0.82)	<0.001	50/92 (1.8)	–
*Anopheles culicifascies*	0 (0.0)	0	0	ND	ND	50/0 (0.0)	–
*Aedes spp.*	13(1.0)	5	8	0.61 (0.32-0.86)	0.43	50/13 (0.2)	–
Dhading district							
*Culex quinquefasciatus*	1050 (93.3)	160	890	0.85 (0.82-0.87)	<0.001	50/1050 (21)	890 (84.8)
*Culex Vishnui*	24 (2.1)	8	16	0.67 (0.45-0.84)	0.042	50/24 (0.5)	–
*Armigeres spp.*	42 (3.7)	15	27	0.64 (0.48-0.78)	0.016	50/42 (0.8)	–
*Anopheles culicifascies*	1 (0.1)	0	1	1.0 (0.05-1.0)	1	50/1 (0.02)	–
*Aedes spp.*	8 (0.7)	3	5	0.63 (0.24-0.91)	0.62	50/8 (0.2)	–
Bara district							
*Culex quinquefasciatus*	575 (83.9)	50	525	0.91 (0.89-0.93)	<0.001	40/575 (14.4)	525 (91.3)
*Culex Vishnui*	45 (6.6)	12	33	0.73 (0.58-0.85)	<0.001	40/45 (1.1)	–
*Armigeres spp.*	57 (8.3)	22	35	0.61 (0.48-0.74)	0.024	40/57 (1.4)	–
*Anopheles culicifascies*	3 (0.4)	1	2	0.67 (0.09-0.99)	1	40/3 (0.1)	–
*Aedes spp.*	5 (0.7)	2	3	0.60 (0.15-0.95)	1	40/5 (0.1)	–
Mahottari district							
*Culex quinquefasciatus*	1155 (90.6)	205	950	0.82 (0.80-0.84)	<0.001	40/1155(28.9)	950 (82.3)
*Culex Vishnui*	45 (3.5)	11	34	0.76 (0.60-0.87)	<0.001	40/45 (1.1)	–
*Armigeres spp.*	65 (5.1)	22	43	0.66 (0.53-0.77)	<0.001	40/65 (1.6)	–
*Anopheles culicifascies*	2 (0.2)	0	2	1.0 (0.22-1.0)	0.33	40/2 (0.1)	–
*Aedes spp.*	8 (0.6)	3	5	0.63 (0.24-0.91)	0.62	40/8 (0.2)	–

ND: indicates not determined.

#### Dhading district.

A total of 1125 mosquitoes were collected by using CDC gravid traps, of which 1050 (93.3%) were *Culex quinquefasciatus*, 75 (6.7%) were non-filarial other mosquitoes such as *Culex vishnui*, *Culex pseudo vishnui,* and *Anopheles* mosquitoes were identified. *Culex quinquefasciatus* mosquitoes were checked for parity in the laboratory, and 890 parous vectors were examined under a microscope for infection with *W. bancrofti*. None of the examined mosquitoes found infection with *W. bancrofti* larvae of any stage (Table 3).

#### Bara district.

A total of 685 mosquitoes were collected using CDC gravid traps, of which 575(83.9%) were *Culex quinquefasciatus,* 110(16.1%) were non-filarial other mosquitoes, such as *Culex vishnui*, *Culex pseudo vishnui,* and *Anopheles* mosquitoes were identified. Filarial mosquitoes were dissected for parity in the laboratory, and 525 parous vectors were examined under a microscope for infection with *W. bancrofti*. None of the examined mosquitoes were found to be infected with *W. bancrofti* larvae of any stage (Table 3).

#### Mahottari district.

A total of 1275 mosquitoes were collected using CDC gravid traps, of which 1155(90.6%) were *Culex quinquefasciatus*, 110(16.1%) were non-filarial other mosquitoes such as *Culex vishnui*, *Culex pseudo vishnui,* and *Anopheles* mosquitoes were identified. Filarial mosquitoes were dissected for parity in the laboratory, and 950 parous vectors were examined under a microscope for infection with *W. bancrofti*. None of the examined mosquitoes found infection with *W. bancrofti* larvae of any stage (Table 3).

### Trend assessment of LF infection in selected endemic districts of Central Nepal

#### Lalitpur district.

**Fig 2:**
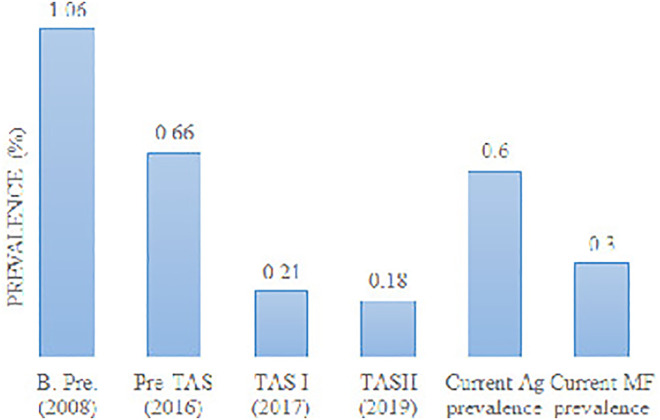
Reported LF prevalence during various stages of assessment in the Lalitpur district of Central Nepal. (B. pre. Baseline prevalence, Ag, antigen, and MF, microfilaria).

A trend analysis of current antigen and MF prevalence with reported baseline survey, pre-TAS, TAS 1, and TAS II seems to show a slight increase in trend (Fig 2).

#### Dhading district.

**Fig 3:**
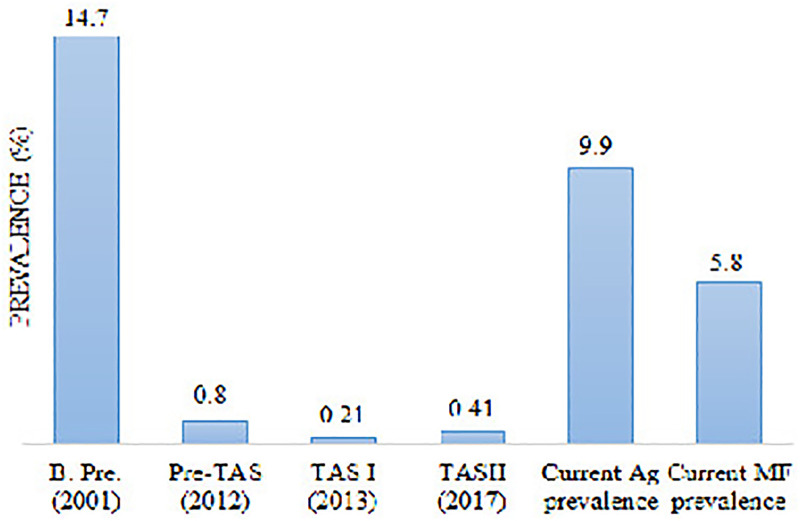
Reported LF prevalence during various stages of assessment in the Dhading district of Central Nepal. (B. pre. Baseline prevalence, Ag, antigen and MF, microfilaria).

Trend analysis of LF infection with baseline prevalence, pre-TAS, TAS1, TAS11 with current antigen and MF prevalence showed a significant increase in trends, which is greater than the critical cut-off value of WHO recommended to stop the MDA program (Fig 3).

#### Bara district.

**Fig 4:**
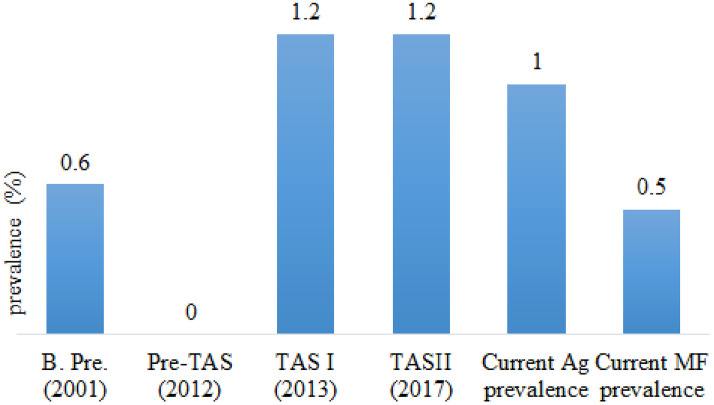
Reported LF prevalence during various stages of assessment in the Bara district of Central Nepal. (B. pre. Baseline prevalence, Ag, antigen and MF, microfilaria).

Trend analysis of LF infection with baseline prevalence, pre-TAS, TAS1, TAS11 with current antigen and MF prevalence showed a significant decrease in trends, which less than the critical cut-off value of the WHO is recommended to stop the MDA program (Fig 4).

#### Mahottari district.

**Fig 5:**
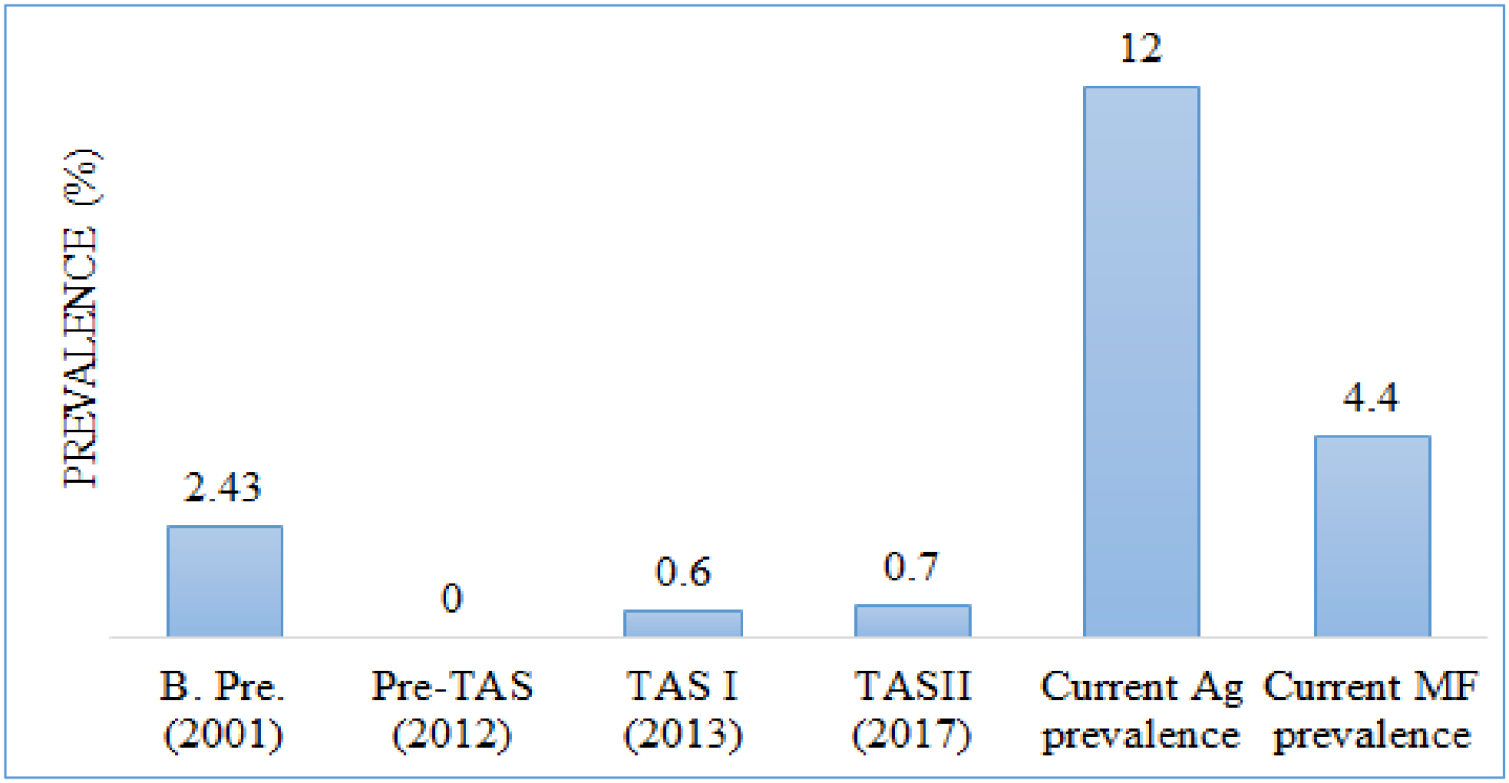
Reported LF prevalence during various stages of assessment in the Mahottari district of Central Nepal. (B. pre. Baseline prevalence, Ag, antigen and MF, microfilaria).

Trend analysis of LF infection with baseline prevalence, pre-TAS, TAS1, TAS11 with current antigen and MF prevalence showed a significant increase in trends, which is greater than the critical cut-off value of WHO recommended to stop the MDA program (Fig 5).

## Discussion and conclusion

Lymphatic filariasis is still endemic in nearly seven districts of Nepal, even after the launch of the MDA program in 2003 with the use of DEC and albendazole. The Nepal Government aimed to eliminate LF by 2020 using recommended MDA coverage of ≥ 65% of the targeted population for 5–6 consecutive years [1], which could interrupt the transmission of LF, but due to unexpected failure of elimination, the aim of the program has been extended up to 2030 to eliminate. Nepal realigned the target of LF elimination by 2030, in line with the global goal. The present study in hotspots showed persistence of CFA infection in children in the age class 5–14 years above the threshold of 2% in two of the four districts evaluated. These four districts cleared all three TASs. These hotspots were identified from the results of TAS1 in the study districts. These hotspots can be the monitoring sites for infection in the post-validation phase.

Further analysis showed high LF prevalence in areas with high baseline prevalence and low MDA rounds, which could be correlated with our previously published data of high MDA compliance [10]. However, in some studies, it was also recommended that areas with higher baseline prevalence and MDA compliance would require more MDA rounds to interrupt the transmission of LF. MDA compliance rates are the major factor for the program’s success [17–21]. However, the present study showed that assessed MDA coverage is not significantly correlated with current levels of LF infection. The assessed MDA coverage data may pose some uncertainty with the coverage estimates.

We investigated microscopy-based assessment of vector infection/ infectivity for LF in hotspots of selected endemic districts of Central Nepal. Various survey studies have been carried out during this study, reported CFA prevalence rates ranging from 0.6% to 12%. Similarly, our previously published data of MF prevalence ranges from 0.3% to 5.8% [10] in hotspots of the selected four endemic districts. However, xenomonitoring showed none of the vector mosquitoes were positive for infection. Our examination for infectivity also showed negative [22,23]. Some of the studies showed that mosquito infection/ infectivity was very low in xenomonitoring, but using molecular xenomonitoring, which can demonstrate active transmission [24,25]. The present study showed that xenomonitoring by microscopy is not sensitive enough to capture signals in areas with residual infection and Mf carriers.

The present study showed persistence of infection above the threshold in hotspots. Earlier reports showed persistent Mf carriers, which can be a source of infection for further transmission. These sites can be followed up for assessing infection, and the results can be used for making a decision on conducting two more rounds of MDA in the hotspots. With the advent of the triple drug regimen, additional rounds of MDA can consider to be use with the triple drug. Studies showed triple-drug treatment using ivermectin, DEC, and albendazole (IDA) is highly efficient for the long-term effect of clearing microfilaremia of W. bancrofti [26–28].

## Supporting information

S1 Fig A positive test result at 10 minutes. (TIF)

S2 Fig Night blood collection by finger finger-prick method for the MF study in the field. (TIF)

S3 Fig Blood smear staining in the laboratory. (TIF)

S4 Fig A patient with chronic clinical manifestation (elephantiasis). (TIFF)

S5 Fig A collection of mosquitoes by a gravid trap. (TIF)

## References

[1] WHO. Lymphatic filariasis: a handbook of practical entomology for national lymphatic filariasis elimination programs. 2013. https://www.who.int/publications/i/item/9789241505642

[2] Nutman TB. Insights into the pathogenesis of disease in human Lymphatic Filariasis. Lymphatic Research and Biology. 2013;11(3):144–8. doi: 10.1089/lrb.2013.0021 24044755 PMC3780283

[3] Local Burden of Disease 2019 Neglected Tropical Diseases Collaborators. The global distribution of lymphatic filariasis, 2000-18: a geospatial analysis. Lancet Glob Health. 2020;8(9):e1186–94. doi: 10.1016/S2214-109X(20)30286-2 32827480 PMC7443698

[4] WHO. Lymphatic filariasis. 2023. [Cited 2024 March 16]. https://www.who.int/news-room/fact-sheets/detail/lymphatic-filariasis

[5] WHO. Global program to eliminate lymphatic filariasis: progress report. Weekly Epidemiological Record. 2024;99(40):565–76.

[6] WHO. Lymphatic Filariasis–Key Facts. 2024. [Cited 2024 November]. https://www.who.int/news--room/factsheets/detail/lymphatic-filariasis

[7] WHO. Ending the neglect to attain the Sustainable Development Goals: a road map for neglected tropical diseases 2021–2030. 2020. https://www.who.int/publications/i/item/9789240010352

[8] Vos T, Lim SS, Abbafati C, Abbas KM, Abbasi M, Abbasifard M, et al. Global burden of 369 diseases and injuries in 204 countries and territories, 1990–2019: a systematic analysis for the Global Burden of Disease Study 2019. The Lancet. 2020;396(10258):1204–22. doi: 10.1016/S0140-6736(20)30925-9 PMC7567026 33069326

[9] WHO. Towards eliminating lymphatic filariasis: progress in the South-East Asia region. WHO Regional Office for South-East Asia; 2013. https://www.who.int/publications/i/item/sea-cd-266

[10] Mehta PK, Maharjan M. Assessment of microfilaremia in “hotspots” of four lymphatic filariasis endemic districts of Nepal during post-MDA surveillance. PLoS Negl Trop Dis. 2024;18(1):e0011932. doi: 10.1371/journal.pntd.0011932 38295107 PMC10861036

[11] Ojha CR, Joshi B, Kc KP, Dumre SP, Yogi KK, Bhatta B, et al. Impact of mass drug administration for elimination of lymphatic filariasis in Nepal. PLoS Negl Trop Dis. 2017;11(7):e0005788. doi: 10.1371/journal.pntd.0005788 28723904 PMC5536438

[12] Department of Health Services. Annual report 2017–2018. Department of Health Services. [Cited 17 March 2023] https://dohs.gov.np/annual-report-2074-75/

[13] Department of Urban Development and Construction. [Cited 25 March 2024]. http://www.dudbc.gov.np

[14] WHO. The role of polymerase chain reaction techniques for assessing lymphatic filariasis transmission. 2009. [Cited 21 March 2024]. https://www.who.int/publications/i/item/WHO-HTM-NTD-PCT-2009.1

[15] Irish SR, Moore SJ, Derua YA, Bruce J, Cameron MM. Evaluation of gravid traps for the collection of Culex quinquefasciatus, a vector of lymphatic filariasis in Tanzania. Trans R Soc Trop Med Hyg. 2013;107(1):15–22. doi: 10.1093/trstmh/trs001 23222942

[16] Pedersen EM, Stolk WA, Laney SJ, Michael E. The role of monitoring mosquito infection in the Global Programme to Eliminate Lymphatic Filariasis. Trends Parasitol. 2009;25(7):319–27. doi: 10.1016/j.pt.2009.03.013 19559649

[17] Guideline. Alternative mass drug administration regimens to eliminate lymphatic filariasis. World Health Organization; 2017. 29565523

[18] Lahariya C, Mishra A. Strengthening of mass drug administration implementation is required to eliminate lymphatic filariasis from India: an evaluation study. J Vector Borne Dis. 2008;45(4):313–20. 19248659

[19] Michael E, Malecela-Lazaro MN, Simonsen PE, Pedersen EM, Barker G, Kumar A, et al. Mathematical modelling and the control of lymphatic filariasis. Lancet Infect Dis. 2004;4(4):223–34. doi: 10.1016/S1473-3099(04)00973-9 15050941

[20] Norman RA, Chan MS, Srividya A, Pani SP, Ramaiah KD, Vanamail P, et al. EPIFIL: the development of an age-structured model for describing the transmission dynamics and control of lymphatic filariasis. Epidemiol Infect. 2000;124(3):529–41. doi: 10.1017/s0950268899003702 10982078 PMC2810940

[21] Plaisier AP, Stolk WA, van Oortmarssen GJ, Habbema JD. Effectiveness of annual ivermectin treatment for Wuchereria bancrofti infection. Parasitol Today. 2000;16(7):298–302. doi: 10.1016/s0169-4758(00)01691-4 10858649

[22] Krentel A, Damayanti R, Titaley CR, Suharno N, Bradley M, Lynam T. Improving coverage and compliance in mass drug administration for the elimination of LF in Two “Endgame” Districts in Indonesia using micronarrative surveys. PLoS Negl Trop Dis. 2016;10(11):e0005027. doi: 10.1371/journal.pntd.0005027 27812107 PMC5094770

[23] Shuford KV, Turner HC, Anderson RM. Compliance with anthelmintic treatment in the neglected tropical diseases control programmes: a systematic review. Parasit Vectors. 2016;9:29. doi: 10.1186/s13071-016-1311-1 26813098 PMC4729159

[24] Bartilol B, Babu L, Garama K, Karisa J, Kamau A, Mwandawiro C, et al. Xenomonitoring of Lymphatic filariasis and risk factors for transmission on the Kenyan coast. Cold Spring Harbor Laboratory; 2024. doi: 10.1101/2024.01.23.24301642

[25] Njenga SM, Kanyi HM, Mwatele CM, Mukoko DA, Bockarie MJ, Kelly-Hope LA. Integrated survey of helminthic neglected tropical diseases and comparison of two mosquito sampling methods for lymphatic filariasis molecular xenomonitoring in the River Galana area, Kilifi County, coastal Kenya. PLoS One. 2022;17(12):e0278655. doi: 10.1371/journal.pone.0278655 36490233 PMC9733851

[26] King CL, Suamani J, Sanuku N, Cheng Y-C, Satofan S, Mancuso B, et al. A Trial of a triple-drug treatment for Lymphatic Filariasis. N Engl J Med. 2018;379(19):1801–10. doi: 10.1056/NEJMoa1706854 30403937 PMC6194477

[27] King CL, Weil GJ, Kazura JW. Single-Dose Triple-Drug Therapy for *Wuchereria bancrofti* - 5-Year Follow-up. N Engl J Med. 2020;382(20):1956–7. doi: 10.1056/NEJMc1914262 32402169 PMC7175637

[28] Supali T, Djuardi Y, Christian M, Iskandar E, Alfian R, Maylasari R, et al. An open label, randomized clinical trial to compare the tolerability and efficacy of ivermectin plus diethylcarbamazine and albendazole vs. diethylcarbamazine plus albendazole for treatment of brugian filariasis in Indonesia. PLoS Negl Trop Dis. 2021;15(3):e0009294. doi: 10.1371/journal.pntd.0009294 33780481 PMC8031952

