# Supplementary figures and images for: Transmission status of lymphatic filariasis in hotspots of filarial infection, persistent districts of nepal during post-MDA surveillance
Source: PLoS One. 2026 Jan 16;21(1):e0338141. doi: 10.1371/journal.pone.0338141 (PMC12810804; doi:10.1371/journal.pone.0338141)

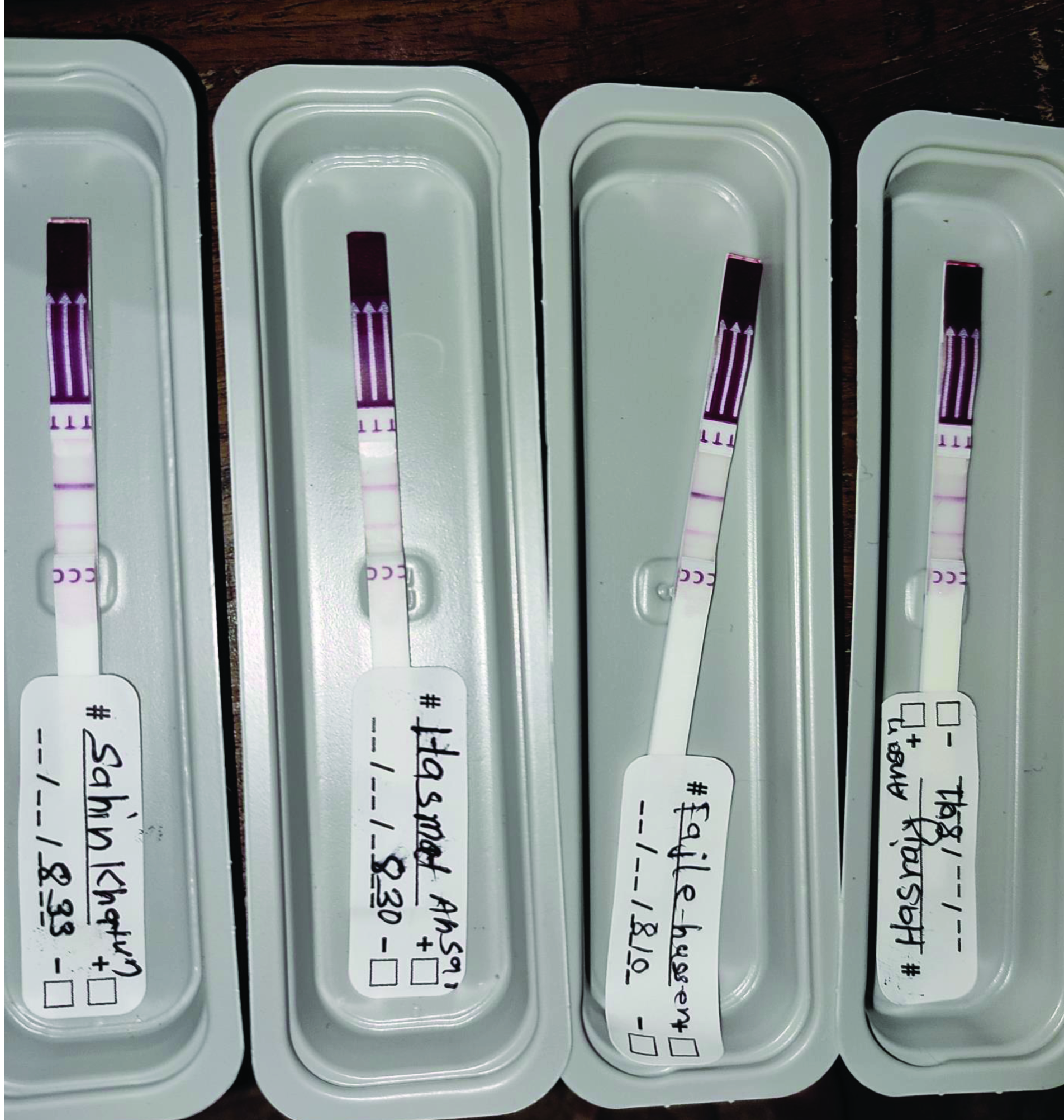

Supplement: S1 Fig — (TIF) [file pone.0338141.s001.tif]

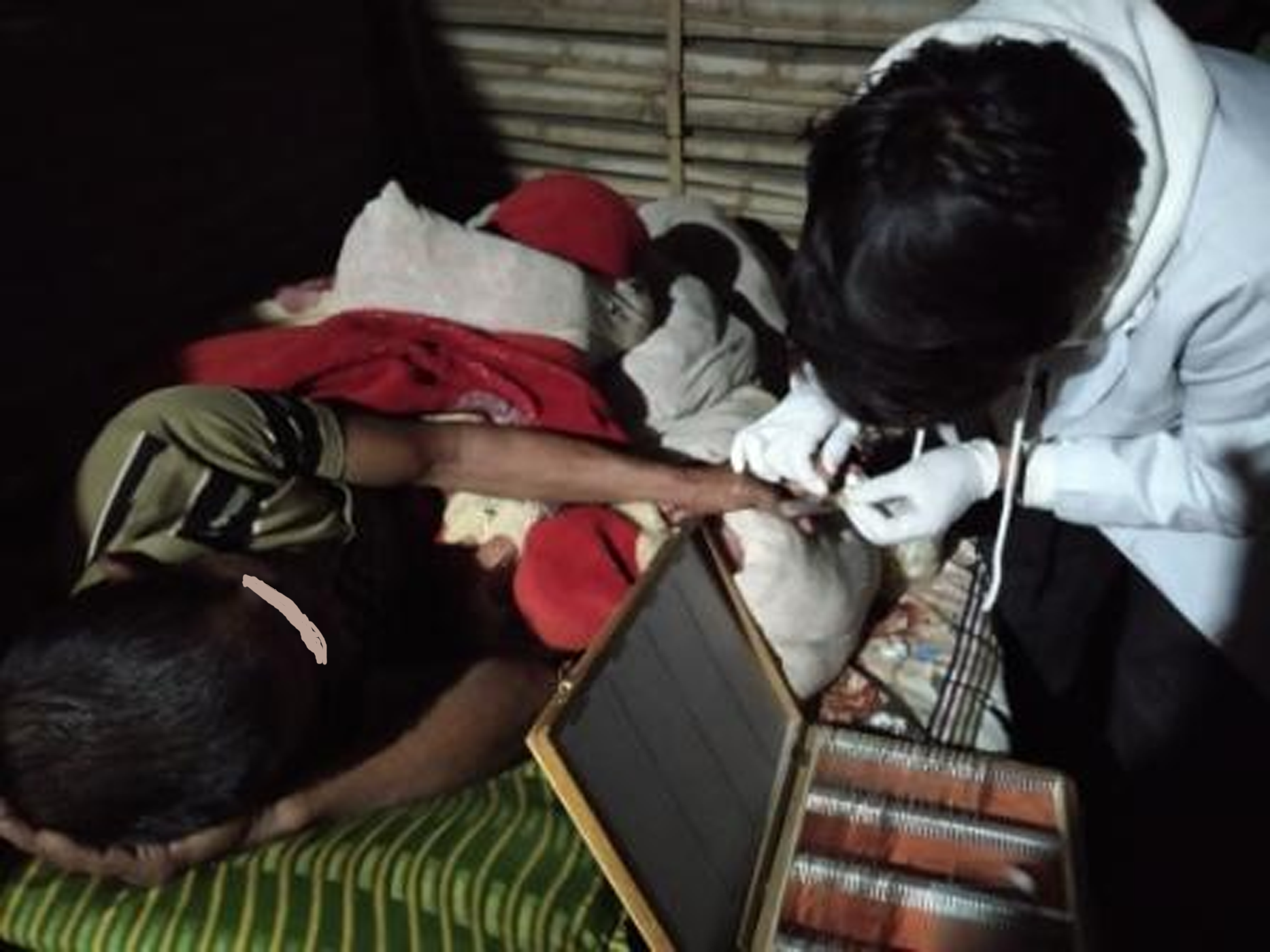

Supplement: S2 Fig — (TIF) [file pone.0338141.s002.tif]

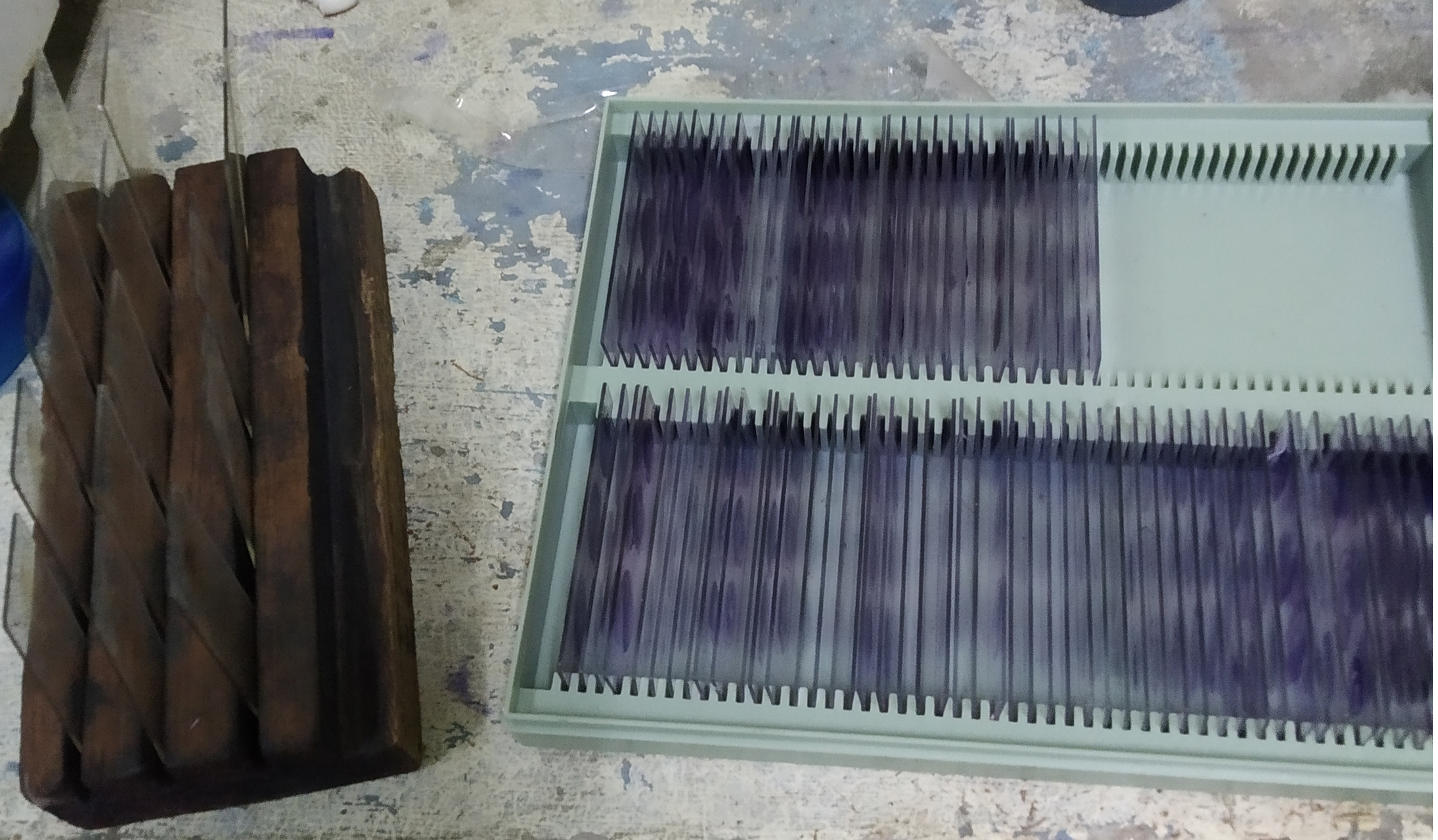

Supplement: S3 Fig — (TIF) [file pone.0338141.s003.tif]

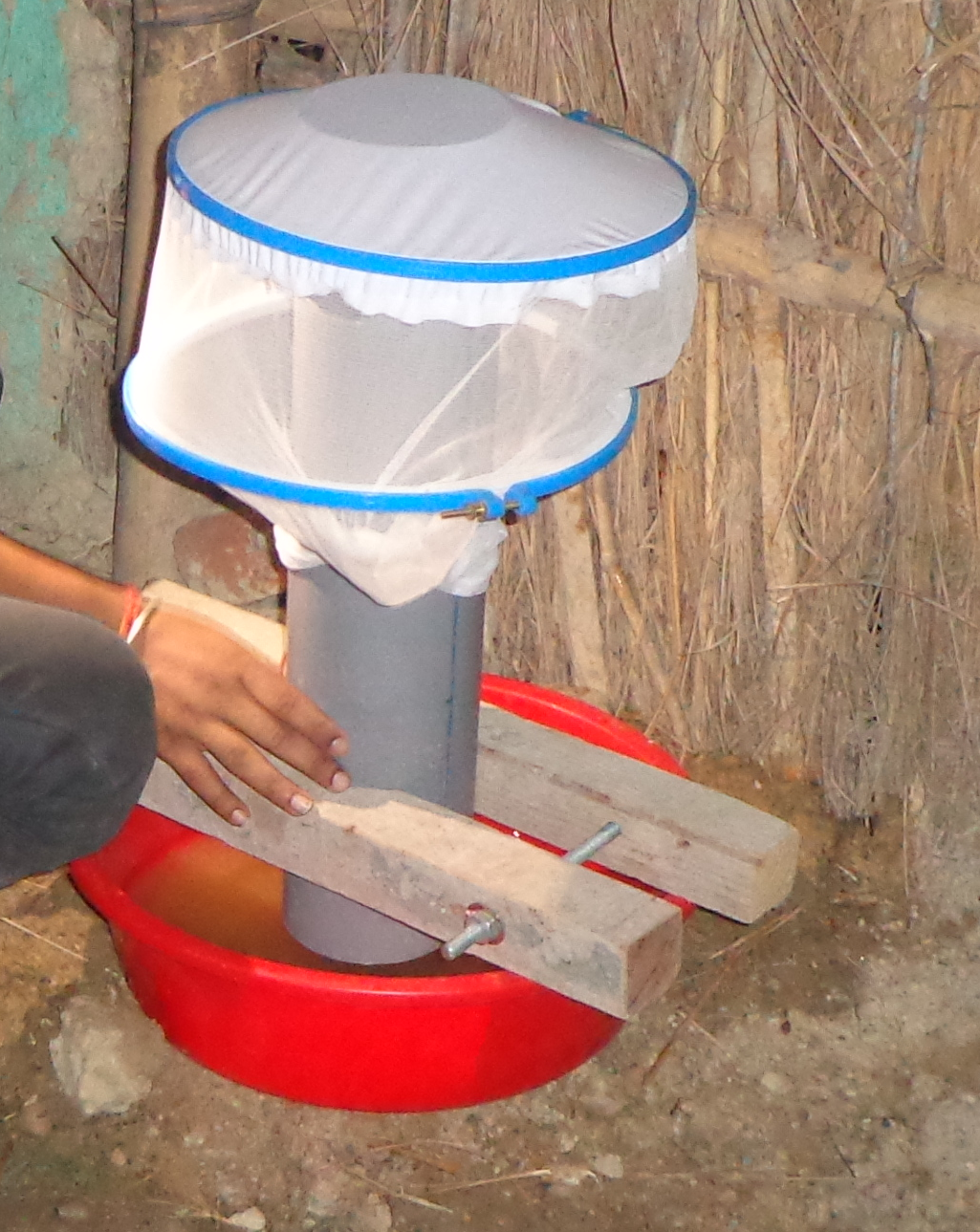

Supplement: S5 Fig — (TIF) [file pone.0338141.s005.tif]
